# How Much Asthma Is Atopic in Children?

**DOI:** 10.3389/fped.2017.00122

**Published:** 2017-05-26

**Authors:** Pasquale Comberiati, Maria Elisa Di Cicco, Sofia D’Elios, Diego G. Peroni

**Affiliations:** ^1^Department of Surgical Sciences, Dentistry, Gynecology and Pediatrics, Pediatric Clinic, University of Verona, Verona, Italy; ^2^Department of Clinical and Experimental Medicine, Section of Pediatric, University of Pisa, Pisa, Italy

**Keywords:** allergy, asthma, atopy, children, phenotype, endotype, wheezing

## Introduction

Asthma is the most common chronic childhood disease worldwide and poses a significant health and socioeconomic burden. The prevalence of this disease differs geographically and seems on the rise in many parts of the world. (1).

Cross-sectional and longitudinal studies have identified several indicators associated with high risk of asthma (2). Personal history of atopy in early life seems to be one of the key factors of an individual’s risk of persistent asthma. Indeed, numerous studies have demonstrated that early and multiple allergic sensitization (AS) specific to aeroallergens and some food antigens places a strong risk for the development of asthma in children (2–6).

Atopic asthma represents the most common form of asthma in the pediatric age and is characterized by eosinophilic airway inflammation associated with specific immunoglobulin E (IgE) antibodies sensitization to various allergens, as evidenced by serology or skin prick test (7). According to a large population survey, including data from people aged 6–59 years (the Third National Health and Nutrition Examination Survey), 56.3% of asthma cases in the United States were attributable to atopy (8). Similarly, the 14-year follow-up analysis of an Australian community-based birth cohort showed that the proportion of asthma associated with atopy was 52% overall (9). The results of a recent cohort study, which followed a large group of newborns over various periods, indicated that the prevalence of asthma from 4 to 16 years is markedly higher among ever-allergic sensitized children compared to never-sensitized ones (10). At a population level, a recently published longitudinal study that prospectively collected data over the first four decades of life showed that the occurrence of asthma before the age of 13 years was more strongly associated with atopy and greater airflow obstruction than later onset asthma (11).

Nevertheless, there is still controversy about the causal relationship between atopy and asthma, as other non-allergenic factors may trigger the specific IgE pathway and influence the occurrence of this disease (12). Over the last 20 years, accumulating evidence has shown that the interrelation between AS and subsequent development of asthma is more complex than a linear dose–response relationship, with gene–environment interactions and epigenetic modifications playing crucial pathophysiological roles (13).

One impediment to understand the relationship between atopy and asthma is the common use of atopy as a binary variable (i.e., sensitized or non-sensitized). Another major impediment is that asthma is a heterogeneous condition, which comprises several different disease endotypes sharing similar symptoms. Recent data indicate that atopy may also encompass distinct endotypes characterized by different patterns of association with asthma (13).

This opinion article outlines the most recent and debated findings about the interrelation between atopy and pediatric asthma.

## The Burden of Atopy in Pediatric Asthma

Atopy is described as the personal tendency to produce IgE antibodies in response to exposure to common allergens, with an increased risk of developing typical diseases such as asthma, rhinoconjunctivitis, or atopic dermatitis. In clinical practice, as in most studies, atopy is often defined either as the presence of serum allergen-specific IgE antibodies or positive skin prick tests. However, a positive allergy test does not necessarily imply clinical reactivity upon allergen exposure. Indeed, a relevant proportion of such defined “atopic” children do not develop any allergy-related disease (10).

There is emerging evidence that both quantification of atopy and timing of onset of AS provide more valuable information to assess the risk of persistent asthma in children (6).

Indeed, different high-risk cohort studies showed that eczema may be a good predictor of childhood asthma when associated with AS; conversely, the risk of subsequent asthma seemed not to be increased with non-atopic eczema (14, 15).

The development of AS and lower respiratory tract viral infections in the first few years of life seem the most consistent environmental risk factors for subsequent asthma at school age, with sensitization to multiple aeroallergens (especially perennial allergens) and rhinovirus-induced wheezing conferring the greatest risk (16–18). There are several mechanisms by which respiratory viral infections are supposed to interplay with allergic inflammation to lead to airway dysfunction, wheezing, and asthma (19). Of interest, recent data showed that both rhinovirus infections and aeroallergens can induce airway epithelial cells to produce interleukin-33; this cytokine can promote allergic airway inflammation and remodeling (16). Nevertheless, the temporal relationship between atopy and viral wheezing during early life is not fully elucidated. The high-risk birth cohort “Childhood Origins of ASThma (COAST)” study recently showed a sequential relationship whereby AS seems to precede rhinovirus-induced wheezing (20).

Notably, it has become evident that early AS to aeroallergens and respiratory viral infections synergistically enhance both the risk of preschool wheezing and subsequent persistent asthma (17). In a recent extension of the COAST study, children with both inhalants sensitization and rhinovirus-induced wheezing by age 3 years had the highest odds ratios for asthma inception at school age and for the persistence of asthma out to adolescence (17). Interestingly, this study suggested that the timing of AS to aeroallergens in early life can significantly influence the risk of future asthma as children who were sensitized to one or more aeroallergens by age 1 year showed the highest rate of asthma at age 13 years; conversely, if sensitization occurred after age 5 years the risk of asthma at adolescence was not different from that of participants who were not sensitized through 13 years (17).

Increasing data also show that the level of allergen-specific IgE antibodies and the size of skin test wheal to aeroallergens can better help identifying children at risk of preschool wheezing and subsequent asthma than a simple positive allergy test (21–23). Similar quantitative relationship has also been reported in relation to asthma severity and severe asthma exacerbations in children (23, 24). Notably, a recent report suggested that in severe pediatric asthma both allergen-specific IgE antibodies and skin prick tests should be carried out and quantified, as these tests are not always concordant in this specific population of patients (25).

In the pediatric age, there is also evidence of a “quantitative synergism” between aeroallergen-specific IgE levels (e.g., to house dust mite) and respiratory viral infections in enhancing the odds of acute wheezing and asthma exacerbations, with higher levels of specific IgE conferring the greatest risk (26, 27). An indirect support to these findings comes from a recent clinical trial which showed that preseasonal treatment with anti-IgE antibody omalizumab decreased the rates of seasonal virus-induced exacerbations of asthma (28).

Although the association between early aeroallergen sensitization and allergic airway disease is acknowledged, it is less clear whether early life food sensitization influences the risk of subsequent asthma in children. Despite that several birth cohorts reported an association between early food sensitization and asthma, most of these studies neither have addressed this relationship beyond early childhood nor have considered important confounders (such as early life or concurrent eczema and wheezing) (29). Moreover, the number of tested food allergens varied across studies, with egg white and cow’s milk being the most common (29). Recently, two independent prospective studies found that sensitization to food only in the first 2 years of life was associated with increased risk of asthma by age 10–12 years (30). However, both studies defined current asthma based on questionnaire data and were unable to address the relationship between specific food allergen sensitization and subsequent occurrence of asthma. Noteworthy, the greatest risk of asthma was observed in children who had sensitization to both food and aeroallergen by 2 years of life (30).

## Heterogeneity of Atopy and Pediatric Asthma

Currently, the lack of agreement on the definition of asthma, both in clinical practice and research studies, strongly contributes to the complexity in understanding the relationship between atopy and asthma (31).

One major impediment to this process is the mounting recognition that asthma is not a single disease, but a collection of several disease entities, also referred to as endotypes. Although presenting with similar observable clinical characteristics (phenotypes), asthma endotypes seem to arise *via* distinct and unique pathogenic pathways and may also be associated with different genetic predisposition and environmental exposures (31, 32).

This constraint also applies to preschool wheezing illness, which is a heterogeneous condition with several phenotypic expressions and a complex relationship with development of asthma later in life (32). Even though recurrent wheezing is one of the major symptoms of asthma and that asthmatics are more likely than other children to wheeze in childhood, most children who wheeze during early life will have a resolution of the symptom by age 3 years or 6 years at the latest (33). Further impediment relevant to atopic asthma in preschool children is the difficulty in making a clear diagnosis; at this age, spirometry cannot be reliably performed and the diagnosis of asthma is often based on the clinical pattern of wheezing episodes or parental reports of wheezing, which may overestimate the prevalence of this condition (34). To this purpose, during the past two decades, many attempts have been made to characterize childhood wheezing phenotypes and predict their trajectories of transition in asthma. Notably, distinct birth cohort studies showed that atopy is strongly associated with “intermediate and late-onset” wheezing phenotypes, which confer the higher risk of physician-diagnosed asthma at school age and up to adolescence (35–37).

There is growing evidence to show that atopy also comprises distinct endotypes that confer different risks of persistent asthma. Longitudinal follow-up of different birth cohorts across the globe showed that the combination of early and multiple AS represents a strong risk factor for persistent and severe asthma (18, 23, 38).

In a recent British unselected birth cohort study, Simpson and colleagues considered both the type and the time of onset of sensitization to specific allergens in relation to asthma occurrence and severity. Most participants in this cohort with “conventionally” defined atopy were clustered in a completely unsupervised manner into four different atopic classes, named “multiple early,” “multiple late,” “dust mite,” and “non-dust mite” (18); interestingly, only those children sensitized to multiple aeroallergens at an early age reported a significant increase in risk for asthma inception, severe asthma exacerbations leading to admission and impaired lung function ascertained by age 8 years (18). Simpson and coworkers subsequently demonstrated that children belonging to different atopic classes had different environmental exposures, supporting the hypothesis that these classes may correspond to distinct endotypes of atopy (13). More recently, the same group of researchers corroborated all these findings by showing that strikingly similar atopy classes, with similar correlation with clinical outcomes (including asthma and lung function), could be identified in the independent population of children belonging to the Isle of Wight birth cohort (38).

## Conclusion

From a clinical perspective, all these data suggest that AS should not simply be considered an all or nothing phenomenon; rather, the level of allergen-specific IgE and/or the size of skin prick test wheal diameter as well as the timing of occurrence should all be considered when defining the relationship between atopy and pediatric asthma.

Further studies should be designed to identify biological markers able to better characterize wheezing phenotypes in preschool children associated with elevated risk of persistent asthma. Classifying pediatric asthma into more specific clinical phenotypes and biological endotypes is mandatory to define personalized and effective treatment and to target the current elusive goal of primary prevention of asthma.

## Author Contributions

PC wrote the initial draft. All the authors participated in critical revision of the manuscript and provided important intellectual input and approved the final version. All the authors had full access to all of the data and participated in the interpretation of the findings.

## Conflict of Interest Statement

The authors declare that the research was conducted in the absence of any commercial or financial relationships that could be construed as a potential conflict of interest.

## Abbreviations

AS, allergic sensitization; IgE, immunoglobulin E.
